# Widespread endocrine-disrupting pollutant alters male courtship behavior in a livebearing fish

**DOI:** 10.1093/beheco/arag118

**Published:** 2026-09-22

**Authors:** Shiho Ozeki, Anna Renner, Raiko Rafeeq, Jason Beynon-Fankhauser, Jack L Manera, Mirjam Amcoff, Anne Peters, Michael G Bertram, Niclas Kolm, Jake M Martin, Bob B M Wong, John L Fitzpatrick

**Affiliations:** School of Biological Sciences, Monash University, 25 Rainforest Walk, Clayton, Victoria 3800, Australia; Department of Zoology, Stockholm University, Svante Arrhenius väg 18b, Stockholm 114 18, Sweden; School of Biological Sciences, Monash University, 25 Rainforest Walk, Clayton, Victoria 3800, Australia; School of Biological Sciences, Monash University, 25 Rainforest Walk, Clayton, Victoria 3800, Australia; School of Biological Sciences, Monash University, 25 Rainforest Walk, Clayton, Victoria 3800, Australia; Department of Zoology, Stockholm University, Svante Arrhenius väg 18b, Stockholm 114 18, Sweden; School of Biological Sciences, Monash University, 25 Rainforest Walk, Clayton, Victoria 3800, Australia; School of Biological Sciences, Monash University, 25 Rainforest Walk, Clayton, Victoria 3800, Australia; Department of Zoology, Stockholm University, Svante Arrhenius väg 18b, Stockholm 114 18, Sweden; Department of Wildlife, Fish, and Environmental Studies, Swedish University of Agricultural Sciences, Skogsmarksgränd 17, Umeå 907 36, Sweden; Department of Zoology, Stockholm University, Svante Arrhenius väg 18b, Stockholm 114 18, Sweden; School of Biological Sciences, Monash University, 25 Rainforest Walk, Clayton, Victoria 3800, Australia; School of Life and Environmental Sciences, Deakin University, 75 Pidgons Road, Waurn Ponds, Victoria 3216, Australia; School of Agriculture, Biomedicine and Environment, La Trobe University, Corner Plenty Road and Kingsbury Drive, Bundoora, Victoria 3086, Australia; School of Biological Sciences, Monash University, 25 Rainforest Walk, Clayton, Victoria 3800, Australia; Department of Zoology, Stockholm University, Svante Arrhenius väg 18b, Stockholm 114 18, Sweden

**Keywords:** endocrine-disrupting chemical, male mate choice, postcopulatory traits, precopulatory traits, sexual selection

## Abstract

Sexual selection is a potent evolutionary force shaping both precopulatory courtship behavior and postcopulatory sperm traits, with male reproductive effort often adjusted to social and mating context. Endocrine-disrupting contaminants are predicted to alter how males allocate reproductive effort, yet their behavioral effects across different mating contexts remain poorly understood. We examined whether exposure to 17α-ethinylestradiol (EE2; 43 to 1,100 ng/L), a synthetic estrogen used in the contraceptive pill that enters aquatic systems via wastewater effluent, alters male courtship behavior and sperm traits in a freshwater fish (*Dermogenys collettei*). Using assays in which males encountered females either sequentially or simultaneously, we quantified courtship behavior and latency to interact, alongside sperm viability, morphology, and performance. Exposure to high EE2 consistently reduced male mating effort. Exposed males were slower to initiate interactions with females and expressed less courtship behavior in both sequential and simultaneous assays. In contrast, EE2 had no detectable effects on sperm traits, and male preferences for female gravid spot size were unaffected. These findings suggest estrogenic pollution can disproportionately reduce mating effort, revealing a decoupling between behavioral and postcopulatory responses.

## Teaser

Environmental contamination by pharmaceutical pollutants is a growing global concern. We exposed male pygmy halfbeaks, a freshwater fish, to the active ingredient in the contraceptive pill that enters waterways through wastewater. We then tested how exposed and unexposed males behaved when encountering potential mates. Males exposed to higher levels were slower to approach females and performed less courtship. In contrast, sperm traits were unaffected, suggesting that reproductive behavior may be particularly sensitive to estrogenic pollution.

## Introduction

Sexual selection has significant implications for individual fitness, population viability, and species persistence by determining which individuals are able to successfully reproduce (Darwin 1871; Andersson 1994; Janicke et al. 2025). Reproductive success is strongly influenced by precopulatory courtship behavior, whereby courters (often males) invest in displays to attract and secure mates (Darwin 1871; Andersson 1994; Wong and Candolin 2005; Rosenthal 2017). Sexual selection can also occur after mating, for example acting on sperm traits to maximize male success under sperm competition (Birkhead and Pizzari 2002; Simmons and Fitzpatrick 2012). Evidence suggests that female quality can influence how male effort into courtship behavior and sperm quality is deployed (Bonduriansky 2001). For instance, due to a positive size-fecundity relationship, larger females often represent greater reproductive value, meaning that allocating greater courtship effort and/or sperm towards larger females can yield greater reproductive returns (Bonduriansky 2001; Kondo et al. 2020).

Human-mediated pollution can disrupt sexually selected traits, including through endocrine-disrupting chemicals (EDCs), which interfere with hormonal pathways regulating growth, development, and reproduction (Hiller-Sturmhöfel and Bartke 1998). One EDC of particular concern is 17α-ethynylestradiol (EE2), a synthetic estrogen used in the contraceptive pill (Aris et al. 2014). EE2 enters aquatic ecosystems primarily through wastewater effluent, resulting in widespread detection (often <1 to 4 µg/L; Aris et al. 2014; Almeida et al. 2020; Prévot D’alvise et al. 2020; global surface-water median 0.0300 µg/L, 95% CrI 0.0001 to 2.3076 µg/L; Martin et al. 2025). As an estrogen receptor agonist, EE2 can mimic natural estrogens and alter reproductive physiology and behavior in non-target wildlife (Aris et al. 2014; Bondesson et al. 2015; Paterni et al. 2017; Baekelandt et al. 2023).

Given the central role of estrogen signaling in reproductive processes (Nelson and Habibi 2013), there are growing concerns that EE2 has widespread effects on courtship behaviors in fishes. For instance, EE2 exposure can reduce courtship behaviors in sand gobies (*Pomatoschistus minutus*) (Saaristo et al. 2010b) and guppies (*Poecilia reticulata*) (Kristensen et al. 2005). However, relatively few studies have examined whether such effects depend on the context in which potential mates are encountered. Existing studies of EE2 effects on male mate choice have largely used simultaneous choice assays, where males can directly compare multiple females at once (Cram et al. 2019; Saaristo et al. 2019). This contrasts with many natural contexts in which males encounter potential mates sequentially, a difference known to fundamentally alter male mating decisions and allocation of reproductive effort (Head et al. 2015; Schlupp 2018).

Simultaneous encounters are expected to promote stronger male choosiness because females can be directly compared, allowing males to allocate reproductive effort more strategically toward higher-value mates (Wong and Jennions 2003; Wong and Svensson 2009; Barry and Kokko 2010). In contrast, sequential encounters require males to assess females one at a time (Bakker and Milinski 1991), relying on memory and internal acceptance thresholds (Cheng et al. 2014), while also imposing higher opportunity costs if a potential mate is rejected (Wong et al. 2004). Because these more cognitively demanding decision-making processes may be particularly sensitive to EDCs such as EE2, which can alter neural and hormonal pathways underlying behavior (Khatoon et al. 2024; Ramasre et al. 2024), the behavioral consequences of EE2 exposure may be especially pronounced under sequential encounter conditions. In addition to impacting courtship behavior, EE2 has been seen to impair sperm traits such as motility, velocity, and concentration (Montgomery et al. 2014; Oropesa et al. 2015; Qin et al. 2022). However, effects are not always consistent, with some studies reporting increases in sperm velocity (Hashimoto et al. 2009; Greven 2010), indicating that the direction and magnitude of EE2 effects on sperm traits may depend on factors such as exposure concentration, exposure duration, and species.

Accordingly, the aim of this research was to examine whether and how EE2 influenced precopulatory courtship behavior and postcopulatory sperm traits in the pygmy halfbeak (*Dermogenys collettei*). Halfbeaks are a small, viviparous, internally fertilizing fish that inhabit freshwater systems across Southeast Asia (Meisner 2001; Greven 2010), where EE2 can be found in surface waters (Tang et al. 2021). Halfbeaks live in mixed-sex shoals where conspecifics readily engage in courtship behaviors (Greven 2010; Devigili et al. 2021). Halfbeaks display sexual dimorphism in body size, with adult females being larger than males, as well as sexual dichromatism, with adult males being more colorful than females (Greven 2010). Female preference for males is influenced by the amount of red coloration on their fins (Reuland et al. 2020; McNeil et al. 2021), while male mate choice is dependent on the size of the orange female gravid spot, which varies in size with the reproduction cycle (Ogden and Fitzpatrick 2019; Ogden et al. 2020). Given that halfbeaks are likely to be exposed to EE2 in the wild and their male courtship behavior is well described (Greven 2007; Greven 2010), they are an ecologically relevant model species for understanding the effects of EE2 on pre and postcopulatory traits under sexual selection.

Within this framework, we tested whether EE2 alters male courtship effort and sperm performance depending on the context in which potential mates are encountered. Specifically, we compared male responses under sequential versus simultaneous female encounter contexts, which differ in the information available for mate assessment and are known to influence mating decisions (Wong and Svensson 2009). We predicted stronger male choosiness in simultaneous trials (Head et al. 2015), and that EE2 exposure would reduce both courtship effort and sperm performance in male halfbeaks, consistent with reported effects of EE2 on reproductive behavior and sperm traits in other fishes (Saaristo et al. 2009; Saaristo et al. 2010b; Montgomery et al. 2014; Oropesa et al. 2015; Qin et al. 2022). We further predicted that EE2 effects would be more pronounced under sequential encounter conditions (Bakker and Milinski 1991), which require more complex mate assessment, resulting in reduced discrimination and less strategic allocation of courtship effort.

## Materials and methods

All procedures were approved by the Stockholm Animal Research Ethical Board (Dnr 12211-2021; Dnr 16124-2023) and the Biological Sciences Animal Ethics Committee of Monash University (AEC 42796), and complied with all relevant State and Federal legislation in Australia.

This project followed the EthoCRED reporting recommendations for behavioral ecotoxicology studies (Bertram et al. 2025) (for a systematic list of all provided criteria, see Appendix A) and follows the MeRIT recommendation for reporting author contributions (Nakagawa et al. 2023).

### Animal collection and housing

Adult halfbeaks (Fig. 1) of both sexes were obtained from a commercial supplier (Imazo AB), which sourced the fish from the Johor River in Singapore. Halfbeaks primarily exhibit courtship-based mating behavior (Greven 2007), and males appear to monopolize access to females through precopulatory competition (Gonçalves et al. 2026). While males may engage in repeated or persistent courtship attempts (Greven 2007), discrete alternative mating tactics (eg, sneak mating) have not been described for this species. Prior to EE2 exposure, the fish were acclimated to laboratory conditions (12 h light: 12 h dark photoperiod and room temperature of 25 to 27 °C) at Stockholm University, Sweden. Fish (*n* = 180 total) were housed in 10 L (length × width × height; 40 × 25 × 15 cm) tanks and fed an alternating diet of flake food and *Artemia* (general fish husbandry was conducted by SO, AR, RR, and JBF).

**Figure 1:**
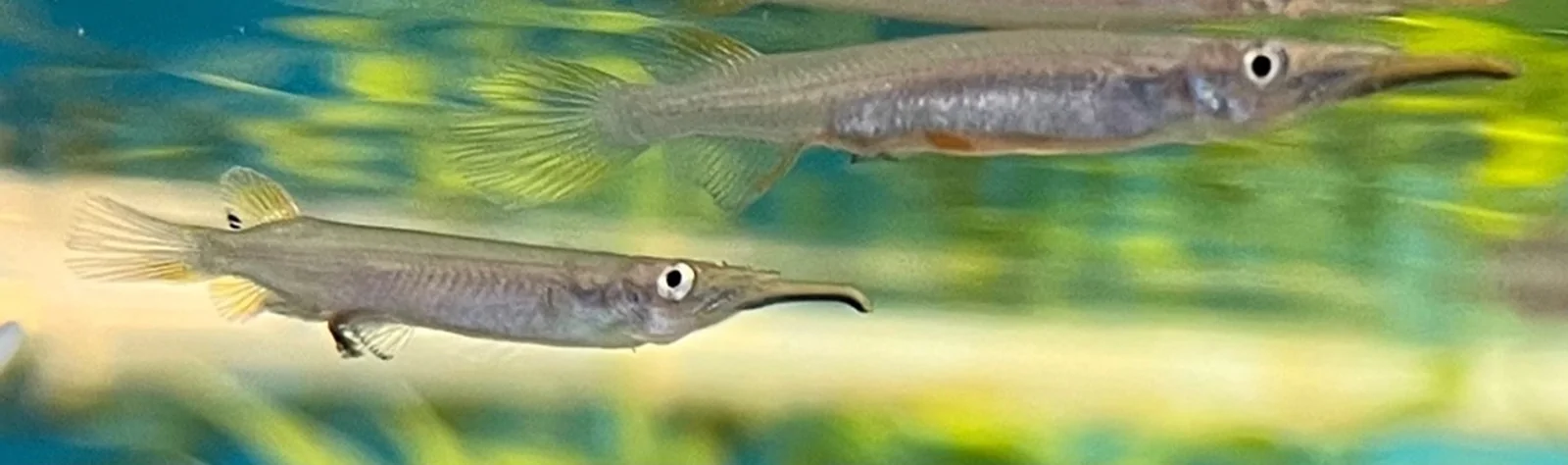
Male pygmy halfbeak (*Dermogenys collettei*) courting a female. The male (left) performs a swimming-under behavior beneath the female (upper right), orienting towards the female's gravid spot (orange abdominal marking).

### Exposure regime

Following the acclimation period and immediately prior to the start of dosing, a single 20% water change was conducted using conditioned freshwater to preserve water quality. Male halfbeaks were then randomly allocated to 1 of 3 treatment groups: a solvent control (0 ng/L), low-dose EE2 (nominal concentration: 30 ng/L), or high-dose EE2 (nominal concentration: 100 ng/L). These concentrations span EE2 levels reported from freshwater systems worldwide (Aris et al. 2014; Martin et al. 2025). A 14 d exposure period was selected based on previous studies demonstrating that this duration is sufficient to elicit morphological, physiological, and behavioral changes in fishes (Saaristo et al. 2010a, 2010b; Lee et al. 2014). Fish were visually size-matched when distributed amongst the exposure tanks to control for any potential body size effects.

During exposure, male fish were housed in 30 tanks (*n* = 10 tanks per treatment), with each tank containing 3 fish (*n* = 30 per treatment). Each tank was filled with conditioned freshwater (90 mL Biotopol [JBL] and 30 g salt per 250 L freshwater), 4 glass cylinders filled with gravel to provide refuge, an air stone, and a printed image of gravel underneath the tank (Schroeder et al. 2014). Two sets of experiments were conducted; one tested the impact of EE2 under a sequential encounter context (detailed in Section 2.2.1), while the other examined a simultaneous encounter (detailed in Section 2.2.2). These 2 experiments used different focal individuals (*n* = 90 per experiment). For both assays, fish were introduced into the exposure system in temporal blocks, with each block corresponding to a single day and including all EE2 treatments. This block structure reflects the staggered introduction of tanks. All individuals experienced a consistent 14 d EE2 exposure period, after which they were tested immediately. Introduction of tanks occurred over 10 blocks (days) for the sequential assay, with 1 tank per treatment introduced per block, and over 5 blocks (days) for the simultaneous assay, with 2 tanks per treatment introduced per block.

During the 14 d exposure period, housing tanks were dosed once daily with EE2 stock solution (by SO, AR and RR), without daily water renewal. This approach was used to maintain EE2 exposure concentrations over time while minimizing repeated disturbance to the fish. The initial stock solution for the exposure was created by dissolving 100 mg of EE2 (CAS: 57-63-6, ≥98%, Merck; E4876) in 200 mL of ethanol (HPLC grade, 95%) to give a concentration of 500,000 μg/L. To prepare the stock solutions for dosing, 0.2 mL of the initial stock solution was mixed with 0.47 mL of ethanol and 999.33 mL of deionized water to create the low EE2 stock solution (100 µg/L). For the high EE2 stock solution (333 µg/L), 0.67 mL of the initial stock solution was combined with 999.33 mL of deionized water. The control stock solution contained 0.67 mL ethanol and 999.33 mL deionized water. Ethanol concentrations were similar across stock solutions, and any differences were negligible following dilution into experimental tanks (stock solution concentrations and preparation methods were calculated by SO and RR). Once the stock solutions were prepared (by AR and JMM), a random code was assigned to each treatment to ensure experimenters remained blind to treatment identity throughout the experiment (code randomized by MA).

The temperature of the housing room (sequential assay: 26.35 ± 0.55 °C, simultaneous assay: 25.93 ± 0.55 °C) and pH (pH ∼ 7) of the exposure tanks were checked consistently throughout the exposure period using a thermometer and pH strips.

### Exposure monitoring and water analysis

Water samples were taken daily (by SO, AR, RR, and JBF) from each tank for chemical analysis to confirm that there was no cross-contamination between treatments and to validate EE2 concentrations. Specifically, using a 50 mL syringe, a 14 mL sample was drawn from each tank. The samples were then stored at −20 °C before being pooled over time such that each tank had one ∼200 mL water sample (60 total samples; 30 per experiment). The water samples were analyzed by the commercial environmental testing company Envirolab Services (MPL Laboratories, Perth; NATA accreditation: 2901; accredited for compliance with ISO/IEC: 17025). Samples were extracted using solid phase extraction (SPE) and quantified by LC-MS/MS, with a limit of quantification (LOQ) of 2 ng/L (see Supplementary Material S1 for detailed chemical analysis methods).

### Behavioral trials

#### Sequential encounter assay

Following the 14 d exposure period, the effect of EE2 on male courtship behavior was examined by analyzing several courtship behaviors in response to 2 stimulus females (assay was conducted by AR). Female receptivity was not directly quantified because female pygmy halfbeaks do not show obvious behavioral cues of receptivity during mating (Greven 2010). To encourage courtship, males were presented with 2 females: one with a small gravid spot (sequential assay: 1.45 ± 0.43 mm^2^, simultaneous assay: 0.98 ± 0.31 mm^2^) and the other with a large gravid spot (sequential assay: 3.63 ± 1.44 mm^2^, simultaneous assay: 2.29 ± 0.68 mm^2^). Female gravid spot size was measured to ensure that the females assigned to the large gravid spot group had a significantly greater gravid spot area compared with the small gravid spot group, whereas body length was kept consistent. To measure the gravid spot, females were placed into a clear plastic photography container (75 × 50 × 25 mm) filled with their housing water. A photo of the gravid spot was captured by placing the photography chamber on top of a plexiglass sheet above a camera (Canon EOS 800D; photographed by AR and RR). Using ImageJ (Schindelin et al. 2012; Rueden et al. 2017), standard body length (mm; see Supplementary Material S3 for detailed length methods, which were identical for males and females) and gravid spot area (mm^2^) were measured from the photographs (by AR, RR and SO). Experimental tanks were filled to a depth of 12 cm with conditioned freshwater (40 × 25 × 15 cm; 24.39 ± 0.80 °C) and equipped with plastic plants and 2 cm of gravel. For each trial, one male was placed on one side of an opaque divider and one female (either with a large or small gravid spot) was placed on the opposite side. The individuals were allowed to acclimate for 1 h. After this acclimation period, the divider was lifted, enabling the male and female to interact freely. Their behavior was then recorded for 20 min (GoPro 12) from the side. During the trial, experimenters left the room to avoid disturbances. Following the first trial, the female was removed, and the male was placed back in the acclimation zone for 15 min whilst the second female (with the opposite-sized gravid spot from the first trial; the order of spot size presentation was randomized) was introduced into the acclimation zone. A second 20 min trial was then recorded. After males had interacted with both females, the fish were returned to holding tanks and the water in the experimental assay tanks was completely drained and refilled to ensure that any conspecific cues from the previous fish did not influence the behavior of the proceeding fish. Male courtship behaviors were subsequently quantified through video recordings using the key-logging tool for quantitative analysis of animal behavior, BORIS (Friard and Gamba 2016). Specifically, the number of swimming under, circling, and mating attempts (Table S1) was recorded. Additionally, the time to first interact was measured by recording the time (s) until the male first displayed a courtship behavior toward a female. Scoring was restricted to a 15 min period following the first observed interaction, or to the end of the video when interactions occurred late in the trial. The videos were scored blind to the female's gravid spot size, and unblinding occurred only after the completion of the scoring, when the identities of the females were revealed at the end of the video. Nine males died during the acclimation and exposure period (control: *n* = 5; low EE2: *n* = 1; high EE2: *n* = 3), leaving 81 males that entered the behavioral trials. A further 5 males were excluded from analyses: 3 control males due to females giving birth during the trial, one low-EE2 male due to injury, and one low-EE2 male due to a female giving birth during the trial. Consequently, a total of 76 males were included in the final analyses. Females were assigned to a unique pair, one with a large gravid spot and the other with a small gravid spot. Some pairs were reused once throughout the course of experiments, thus, to maintain pair ID, each pair of females was isolated and housed in a 5 L (26 × 18 × 12 cm) tank with plastic plants, 2 cm of gravel and an air stone, after their first trial.

#### Simultaneous encounter assay

The simultaneous assay (conducted by SO, RR and JBF) followed the same method as the sequential assay, except the male was presented with both stimulus females simultaneously. As in the sequential assay, the fish were acclimated for 1 h prior to the trial, during which time the male and 2 females were separated by an opaque barrier. After this acclimation period, the barrier was removed, and the fish were allowed to freely interact while the behavior of the male was recorded for 40 min. Male courtship behaviors were scored as described above. A total of 76 trials were conducted, 14 males died during the acclimation and exposure period (control: *n* = 6; low EE2: *n* = 5; high EE2: *n* = 3), and one individual escaped from the acclimation chamber during the behavioral trial and was therefore excluded from behavior analyses.

### Sperm analysis

Males who participated in the simultaneous trial were also used to assess the impact of EE2 on sperm traits (conducted by SO and JBF). Specifically, sperm morphology, viability, and velocity were measured. Protocols for sperm collection and analysis followed Fernlund Isaksson et al. (2022). After the completion of morphological measurements (see Supplementary Material S3), the dead males were washed in deionized water and placed on a glass slide containing ∼1 mL of 9% saline (NaCl) solution. Using a dissection microscope (S9i stereo microscope, Leica Microsystems, Wetzlar, Germany), sperm bundles were then extracted from the male by applying pressure on the abdomen, and were mixed into the saline solution. This mixture (sperm and saline; 10 μL) was transferred into a 1.5 mL microtube with 20 μL of activation solution (Hank's Balanced Salt Solution, Merck; H9394) to activate the sperm for subsequent analyses. The sperm was then kept in an incubator set to 26 °C for 2 min prior to assessing sperm velocity.

Sperm velocity was assessed using an integrated semen analysis system (ISAS, v. 1.2.33, PROiSER R + D, Paterna, Spain), and measured using computer-assisted sperm analysis (CASA) software (×100 magnification; UB 200i Series Microscope and C13-ON camera, PROiSER R + D, Paterna, Spain). The mixture of prepared sperm and HBSS was vigorously mixed before adding 3 μL of the sperm solution to one well of a 12-well multitest slide (MP Biomedical) coated with a 1% polyvinyl alcohol solution. Sperm velocity traits measured included average path velocity (μm/s), straight-line velocity (μm/s), curvilinear velocity (VCL, μm/s), path linearity (%), and proportion of motile sperm (%). The threshold for defining static cells was predetermined at 25 µm/s for VCL, and a minimum of 200 cells per individual were captured.

Sperm viability was measured using a live/dead cell viability kit in which 4 μL of the sperm mixture was combined with 0.5 μL of acridine orange (which stains live cells green) and 0.5 μL of propidium iodide (which stains dead or inviable cells with a loss of membrane integrity red; VitalTest, NordicCell, Denmark) in a new 1.5 mL microtube. After adding the dyes and mixing vigorously, 5 μL of the stained sperm solution was added to a slide with a cover slip and left to incubate for 2 min in the dark. Following the incubation period, photographs of the sperm were taken using UV fluorescence microscopy (×200 magnification; UB 200i Series Microscope and C13-ON camera, PROiSER R + D, Paterna, Spain). For each male, ∼10 photos were taken with ∼20 cells in each photo. Faststone Image Viewer software (2024) was used to convert the photographs and remove blue light. Using the ImageJ cell counter plugin, the dead and live sperm cells were counted (by SO), for a minimum of 3 pairs of photos (each pair of photos had 2 planes of view; one focused on live cells and one focused on dead cells) and 200 cells per individual.

Using the same settings as those used to assess sperm viability but with the backlight on and UV light off (×400 magnification), morphological sperm traits, such as head length, midpiece length, and total length were noted. Tail length was also measured to calculate total sperm length but was not analyzed separately. Approximately 20 to 30 cells were photographed per male, with 5 to 10 cells in each photo. For each male, 5 photos with 5 cells per photo were measured in ImageJ (by JBF).

### Statistical analysis

Statistical analyses were conducted in R (R Core Team 2025) v.4.5.1 using RStudio v.2025.09.1 + 401 (by JLM, JMM, and SO). Bayesian statistical models were fitted using the *brms* (Bürkner 2017) package v.2.23.0 with *CmdStan* v.2.37.0 via *cmdstanr* (Gabry et al. 2025) v.0.8.0. The impact of EE2 exposure was assessed on the following outcomes: the time a male took to first interact with a female (sequential and simultaneous assays), counts of male courtship behavior (sequential and simultaneous assays), preference for female gravid spot size (sequential and simultaneous assays), sperm viability, sperm morphology, and sperm performance (simultaneous assay).

We analyzed traits with Bayesian generalized linear mixed-effects models in *brms*. Time to first interact was modeled using a hurdle-Gamma distribution (log link for the conditional mean and shape parameters and a logit link for the hurdle component). This approach allowed us to model both the probability of interaction and the latency to interact separately. Courtship behavior counts (mating, circling, and swimming under) were modeled with a zero-inflated negative binomial distribution (logit link for the zero-inflation component and log link for the mean and dispersion parameters) and male preference was modeled with a zero-inflated beta-binomial distribution (logit link for the mean and zero-inflation components and log link for the precision parameter). Sperm morphology measurements (head length, midpiece length, total sperm length) were modeled jointly with a multivariate linear mixed effects model, whereas sperm viability (live proportion) and sperm performance (velocity, linearity) were each modeled with univariate linear mixed-effects models.

Fixed effects included EE2 treatment and biologically relevant, trait-specific covariates including male body size, sperm count (for sperm traits), and female presentation order. For preference models, stimulus female covariates were included as relative differences between the 2 females (gravid spot area difference and body size difference). Full model specifications are provided in Supplementary Material S4.

To test whether EE2 concentrations differed among treatments and between experiments, we analyzed measured water concentrations using a linear model in *brms*, with experiment type (simultaneous or sequential assay), treatment, and their interaction as covariates.

All models were fitted with the default priors assigned by *brms*. Convergence was assessed via the Gelman–Rubin statistic (*R̂* < 1.01), effective sample sizes (>1000), and inspection of trace plots. Posterior predictive checks were visually inspected to assess model fit, and model comparison was guided by leave-one-out cross-validation (LOO-CV Vehtari et al. 2017) based on expected log predictive density (ELPD).

Posterior estimates were summarized as marginal means for factor variables and marginal trends for continuous variables, using the *emmeans* (Lenth and Piaskowski 2025) v.1.11.2-8 and *tidybayes* (Kay 2024) v.3.0.7 packages. Pairwise contrasts between treatment groups were derived from posterior draws, and 95% credible intervals (CrIs) were used to evaluate the magnitude and direction of differences. Contrasts were interpreted as providing strong evidence for an effect when the 95% CrI did not overlap zero (or one for odds ratios in the preference model), and as weak or uncertain when CrIs overlapped these reference values.

## Results

### Analytical verification of EE2 concentrations

Analytical verification of EE2 showed that measured EE2 concentrations differed among treatments within each experiment, and between experiments in the high treatment groups (Table 1; for pairwise contrasts see Supplementary Material S5, Table S2). EE2 concentrations were below the detection limit of 2 ng/L in all control tanks (*n* = 10 per experiment).

**Table 1:** Measured concentrations (ng/L) of 17α-ethinylestradiol (EE2) in exposure tanks over the 14 d dosing period for low (*n* = 10 per experiment) and high (*n* = 10 per experiment) EE2 treatments in the sequential and simultaneous encounter assays.

Experiment	Treatment	Mean	Median	SD	Min	Max
Sequential	Low	228	210	43	170	320
Sequential	High	874	870	128	670	1,100
Simultaneous	Low	148	160	60	43	220
Simultaneous	High	564	585	155	260	770

### Reproductive behavior

#### Latency to interact with females

EE2 exposure influenced how long it took males to initiate interactions with females in both experiments. In the sequential encounter experiment, males in both the low and high EE2 treatments initiated interactions later than control males, while the low and high treatments were comparable (Fig. 2, Table 2). In the simultaneous encounter experiment, males in the high EE2 treatment took longer to initiate interactions than males in the control and low EE2 treatments, while the low and control treatments were comparable (Fig. 2, Table 2). For full model results see Supplementary Material S6, Tables S3–S5.

**Figure 2:**
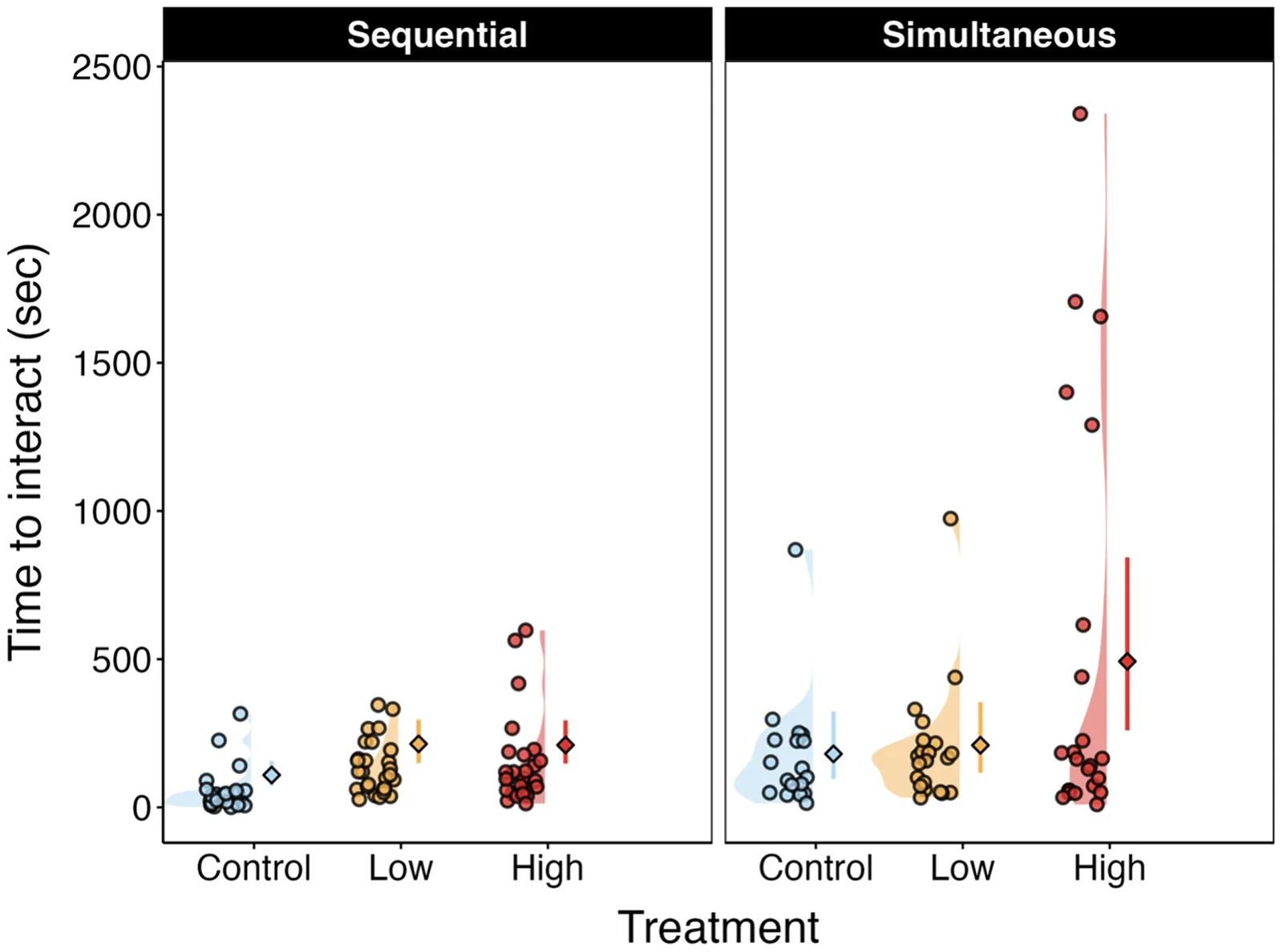
Time taken for males to first interact with a female (sec) under sequential and simultaneous encounter assays, plotted by EE2 exposure treatment. Control males are shown in blue (*n* = 22 sequential assay, 18 simultaneous assay), low EE2 exposure in orange (*n* = 27 sequential assay, 22 simultaneous assay), and high EE2 exposure in red (*n* = 27 sequential assay, 23 simultaneous assay). Colored half-violins represent the kernel density of the observed data, points show jittered raw observations, and solid diamonds indicate model-estimated marginal means with 95% credible intervals.

**Table 2:** Pairwise contrasts of model-estimated means for time taken to first interact across EE2 treatment groups.

Experiment	Contrast	Estimate (s)	Lower 95% CrI	Upper 95% CrI
Sequential	High−control	104.42	25.77	198.40
Sequential	High−low	−3.03	−111.77	104.46
Sequential	Low−control	107.46	27.28	201.78
Simultaneous	High−control	317.80	57.05	663.27
Simultaneous	High−low	285.97	15.17	634.12
Simultaneous	Low−control	31.83	−143.26	210.12

Estimates represent differences in mean latency (s) conditional on interaction, with 95% credible intervals (CrI).

#### Total occurrence of courtship behaviors

EE2 exposure influenced male courtship behavior in both the sequential and simultaneous experiments. In the sequential experiment, males in the high EE2 treatment swam under females less frequently than control males, with low EE2 treatment males showing an intermediate response (Fig. 3). In addition, males performed fewer mating attempts and circling behaviors in the high EE2 treatment compared with controls, again with low EE2 treatments showing intermediate effects.

**Figure 3:**
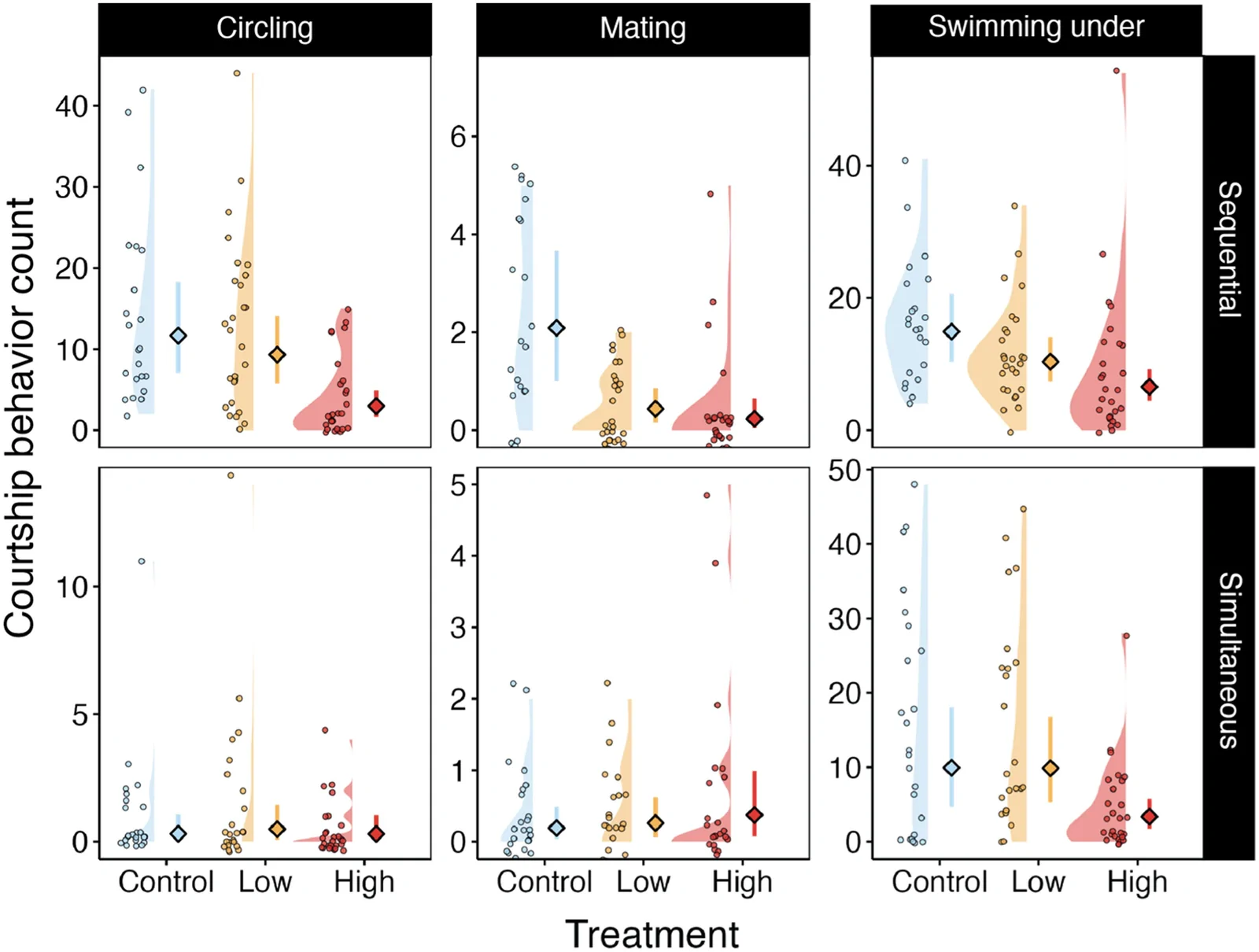
Male courtship behavior counts (circling, mating attempts, and swimming under) during simultaneous and sequential encounter assays, plotted by EE2 exposure treatment. Control males are shown in blue (*n* = 22 sequential assay, 24 simultaneous assay), low EE2 exposure in orange (*n* = 27 sequential assay, 24 simultaneous assay), and high EE2 exposure in red (*n* = 27 sequential assay, 27 simultaneous assay). Colored half-violins depict the kernel density of observed behavior counts, points represent jittered raw observations, and solid diamonds indicate model-estimated marginal means with 95% credible intervals.

In the simultaneous experiment, males in the high EE2 treatment swam under females less frequently than males in the control and low EE2 treatments, whereas mating attempts and circling behavior showed no clear differences among the EE2 treatments (Fig. 3). For full model results, including covariate effects, see Supplementary Material S7, Tables S6–S10.

#### Preference

In both the sequential and simultaneous assays, there was no evidence that EE2 exposure affected male preference for female gravid spot size (Fig. 4). Model-estimated preference values overlapped broadly across treatments in both encounter contexts, and all pairwise treatment contrasts showed substantial uncertainty, with credible intervals overlapping the null expectation. For full model results, including covariate effects, see Tables S11–S12.

**Figure 4:**
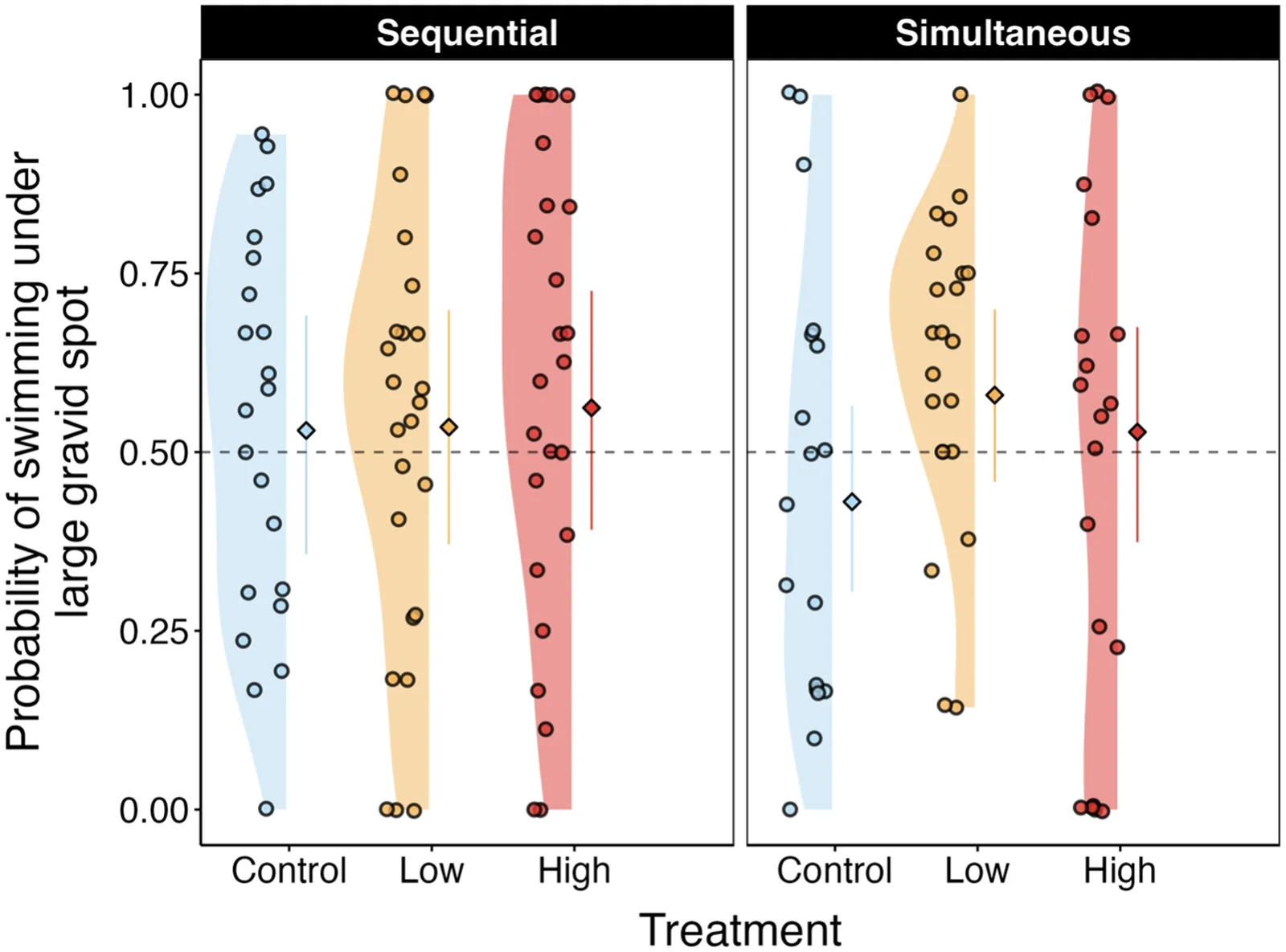
Plot showing probability of swimming under large gravid spot, plotted by exposure treatment. Points above the dotted line represent fish that showed a preference for large gravid spots, and the opposite is true for points below the dotted line. Control fish are represented by blue (*n* = 22 sequential assay, 18 simultaneous assay), low exposure treatment in orange (*n* = 26 sequential assay, 22 simultaneous assay) and high exposure treatment in red (*n* = 25 sequential assay, 21 simultaneous assay). The colored area shows the probability density of the raw data at different values smoothed by a kernel density estimator. Points inside the colored area represent jittered raw data, and solid diamonds are model-estimated marginal means, with error bars showing 95% credible intervals.

### Male sperm traits

EE2 exposure showed no evidence of effects on sperm morphology, viability, or performance (Supplementary Materials S9–S11, Tables S13–S14, and Figures S1–S3).

## Discussion

Our study demonstrates that exposure to the synthetic estrogen EE2 markedly alters male courtship behavior in pygmy halfbeaks, while leaving sperm traits and male mate preferences unaffected. These findings shed light on our current understanding of how EDCs affect the multistage processes of sexual selection, revealing that precopulatory behaviors are more sensitive to short-term EE2 exposure than postcopulatory reproductive traits. Moreover, we show that reductions in male courtship behaviors emerge consistently across both simultaneous and sequential encounter contexts, despite theoretical predictions that mating context should shape male investment strategies (Wong et al. 2004; Barry and Kokko 2010). Taken together, these results highlight the potential for estrogenic pollution to disrupt key behavioral mechanisms underpinning male reproductive behaviors in freshwater fishes.

Although the measured concentrations in our high EE2 treatment exceeded typical surface-water concentrations (Aris et al. 2014), they remain within the range reported from highly contaminated aquatic systems and wastewater-impacted environments worldwide (Tang et al. 2021; Klaic and Jirsa 2022). Accordingly, our high treatment reflects polluted hotspot exposure scenarios rather than average environmental conditions. While exposure at these concentrations may plausibly induce broader physiological effects beyond endocrine disruption, reported acute toxicity thresholds (eg, LC50 values) for fishes exposed to EE2 are generally in the mg/L range, including values of approximately 1.7 mg/L in zebrafish (Versonnen et al. 2003; Schäfers et al. 2007), which is several orders of magnitude higher than those used in the present study. Lower toxicity thresholds have been reported under chronic exposure conditions (Schäfers et al. 2007); however, those estimates were derived from substantially longer exposure durations than the 14 d exposure period used here. Future studies incorporating direct measures of general fish health, such as feeding behavior, baseline activity, or physiological stress markers, alongside internal biomarkers of estrogenic exposure (eg, vitellogenin induction), would further clarify the mechanisms underlying these responses and determine whether the observed behavioral effects are driven primarily by endocrine disruption or broader physiological stress.

The most striking effect of EE2 exposure was a reduction in the initiation and frequency of male courtship behaviors. Males exposed to EE2 took substantially longer to initiate interactions with females in the high-dose treatment in both sequential and simultaneous assays. Male delays in initiating courtship when presented with a female likely have fitness costs, as rival males can instead monopolize access to females (Gonçalves et al. 2026) and/or males may miss the opportunity to court a potential mate. Once interactions with females began, high-dose males performed fewer swimming under behaviors—the most prominent and time-consuming courtship behavior performed by male halfbeaks (Devigili et al. 2021)—in both the simultaneous and sequential assay. We also found some support for reductions in circling behaviors and mating attempts in high-dose males, albeit only when males were exposed to females sequentially.

Because most of the male behaviors that were impacted by EE2 precede and facilitate copulation (Greven 2010; Devigili et al. 2021), reductions in their expression indicates a broad suppression of courtship motivation in male halfbeaks that likely translates into fewer mating opportunities under natural conditions. For example, females may choose males based on courtship duration, vigor, or motor performance (Stoltz et al. 2009; Byers et al. 2010), particularly as male courtship behaviors can advertise male experience (Dukas 2005) and signal male quality (Chargé et al. 2010; Wedell and Kemp 2024) and/or fertility (Weir and Grant 2010). Delayed interactions between the sexes and reduced male courtship effort therefore support the conclusion that EE2 reduces courtship readiness in male halfbeaks and may reduce their ability to secure mating opportunities when reproductive interactions are brief or highly competitive (Devigili et al. 2021). The impact of exposed males on female behavior was not investigated in this study. Because males and females interacted freely during the behavioral trials, variation in female behavior or receptivity may have introduced additional unexplained variation in male courtship motivation. However, because the same stimulus females were used across treatments, variation in female behavior is unlikely to have systematically biased comparisons among EE2 treatment groups, though it may have contributed to unexplained variation in male courtship responses. As female zebrafish have been shown to prefer control males over males exposed to EDCs (Li et al. 2017) and alter their reproductive behavior when paired with EE2 exposed males (Baatrup and Henriksen 2015) further research should disentangle how female halfbeaks react to exposed males and how that may play into male courtship success.

The observed decline in courtship in EE2 exposed males is consistent with reports from other fish species in which EE2 reduces reproductive motivation and courtship behavior (Saaristo et al. 2009), alters gene expression linked to reproductive behavior and sex-specific developmental pathways (Selmoni et al. 2019; Saaristo et al. 2021), and induces behavioral and physiological changes that carry fitness and population-level costs (Bell 2004; Kidd et al. 2007). The role of estrogen in affecting male fertility is well-established in mammals but research on its effects in teleost fishes remains limited (Tang et al. 2017). However, testicular androgen has been established as playing a key role for male sexual behaviors, and hormones from the endocrine system are known to regulate reproductive behaviors (Munakata and Kobayashi 2010). As such, externally introduced chemicals, like EE2, that interfere with hormonal balance, potentially suppress the effects or production of androgens, upregulate estrogen-responsive genes and thereby reduce male reproductive behaviors (Munakata and Kobayashi 2010; Lee et al. 2014; Qin et al. 2022). This potential hormonal mechanism aligns with the reduced courtship behaviors we observed, particularly in high-dose males. Nevertheless, our findings contrast with cases in which EE2 increased male display intensity, as seen in guppies (Saaristo et al. 2021), suggesting that the direction of behavioral change may depend on species-specific endocrine sensitivity, mating system characteristics, and/or exposure regime.

Despite reducing overall courtship effort, EE2 exposure did not alter the strength of male preference in either simultaneous or sequential mating contexts. Theoretical expectations suggest stronger male choosiness under simultaneous than sequential encounters, because simultaneous presentation allows direct comparison among females whereas sequential assessment requires information integration across time (Barry and Kokko 2010). Yet, whether males were presented with 2 females differing in gravid spot size simultaneously, or encountered females with different gravid spot sizes sequentially, the results were the same: male courtship effort was not directed preferentially towards females with larger gravid spots. These findings were surprising as it has previously been demonstrated that male halfbeaks prefer females with larger gravid spots (Ogden et al. 2020). In the present study, female halfbeaks were paired in large versus small gravid spot dyads based solely on the size of the female's gravid spot. In contrast, Ogden et al. (2020) paired female halfbeaks with large versus small gravid spots at different points in their monthly reproductive cycle. This suggests that visual cues alone (ie, the size of the gravid spot) may be insufficient to influence male courtship and mating decisions in the absence of other cues (eg, chemical cues associated with the female's reproductive cycle). Disentangling the cues that influence male mating decisions in halfbeaks is a necessary next step.

In contrast with the reduction in courtship behaviors we detected, we found no evidence that sperm viability, morphology, or swimming performance were affected by EE2 exposure. However, credible intervals around some sperm trait estimates were relatively broad, meaning that more subtle effects cannot be excluded. These results stand in contrast with reports of impaired sperm traits in several other fish species exposed to estrogenic contaminants (Nielsen and Baatrup 2006; Hashimoto et al. 2009; Montgomery et al. 2014; Oropesa et al. 2015; Qin et al. 2022). One possible explanation for this discrepancy is that our 14 d exposure may not have been long enough to affect spermatogenesis substantially. In particular, halfbeaks package sperm in bundles and continuously replenish sperm stores (Greven 2010), which could buffer against rapid disruption of sperm traits and may require longer exposure times—potentially, during development—to reveal effects of EE2 on male reproductive physiology. Nevertheless, the decoupling of behavioral and sperm responses suggests that precopulatory processes may be more immediately sensitive than postcopulatory traits to estrogenic interference.

Broadly, these findings suggest that EDCs may alter the operation of sexual selection by affecting precopulatory behaviors more strongly than postcopulatory traits. Reduced courtship effort could weaken precopulatory sexual selection by lowering male competition for mates and limiting opportunities for strategic reproductive investment (Gonçalves et al. 2026). The absence of detectable effects on male mate preference and sperm traits further suggests that not all components of sexual selection are equally sensitive to short-term EE2 exposure. Given the ubiquity of estrogenic pollutants in freshwater ecosystems, such behavioral disruptions may have far-reaching consequences for reproductive success, population dynamics, and the evolution of mating systems.

## Supplementary Material

arag118_Supplementary_Data

## Data Availability

Analyses reported in this article can be reproduced using the data provided by Ozeki et al. (2026).
