# Supplementary material for: Widespread endocrine-disrupting pollutant alters male courtship behavior in a livebearing fish
Source: Behav Ecol. 2026 Sep 22;37(6):arag118. doi: 10.1093/beheco/arag118 (PMC13658919; doi:10.1093/beheco/arag118)
Supplement: Supplementary file 1 [file arag118_supplementary_data.zip › Ozeki_BE_Supporting-Information.docx]

**Supporting Information for**

**Widespread endocrine-disrupting pollutant alters male courtship behavior in a livebearing fish**

**This Supporting Information includes:** (i) methods for EE2 chemical detection and additional methodological details (sperm count, male length measurements, and statistical model specifications), and (ii) supplementary results, tables, and figures supporting analyses of measured EE2 concentrations, male latency to interact, courtship behavior counts, female preference, and sperm traits (morphology, viability, and performance).

**Supporting methods**

**S1. Method of 17α-ethinylestradiol (EE2) detection**

The calibration standards used to quantify the concentrations of 17α-ethinylestradiol (EE2) were fully extracted and treated identically to EE2 by a method called isotope dilution extraction. This is where labelled internal standards of the target compound are also added at the start of the extraction. This improves the method as these standards/isotopes correct for any losses in the extraction and concentration steps needed to achieve the trace levels required.

The samples were extracted using solid phase extraction (SPE) using cartridges purchased from Biotage – EVOLUTE EXPRESS ABN (part number: 610-0010-BXG or equivalent). After placing the SPEs on a Vac-Elut, they were conditioned by passing through acetonitrile (3 mL) followed by 10% methanol in ultra-high purity water (3 mL) to waste. Methanol (2 mL) was added to the samples (50 mL) before these were passed through the SPEs, again to waste. The SPEs were dried by applying a vacuum for 1–2 min before eluting off the EE2, the internal standards and the surrogate using 100% methanol (3 mL).

To act as a surrogate standard, 10 µL of 1 µg/mL 17α-trenbolone (17α-hydroxyestra-4,9,11-trien-3-one; CAS: 80657-17-6; Novachem, Germany) in methanol (LC-MS/MS grade, ≥99.9%) was then added to each sample, as well as to all calibration standards. To act as the isotope dilution internal standard, 10 µL of 20 µg/mL ethynylestradiol-2,4,16,16-d4 (CAS: 350820-06-3; Toronto Research Chemicals) in methanol (LC-MS/MS grade, ≥99.9%) was then added to each sample, as well as to all calibration standards. The extract was concentrated to 50 µL using an argon blowdown. To this, 200 µL of ultra-high purity water was added, before samples were analyzed using LC-MS/MS. Injection of ~100 µL was performed in electrospray ionization (ESI) mode onto Zorbax Eclipse Plus C18 (1.8 µm) 50 mm length $\times$ 2.1 mm inner diameter. Three transitions were monitored for EE2 (quantification ion: 295 → 159.1, collision energy: 33V; confirmatory ion: 294.9 → 199.1, collision energy: 40V; secondary confirmatory ion: 295.0 → 145.2, collision energy: 42V) and the 17α-trenbolone surrogate standard (quantification ion: 271.2 → 253.4, collision energy –22 V; confirmatory ion: 271.2 → 199.3, collision energy: –25 V). The gradient started at an initial 90% of 0.5 mM ammonium fluoride in water and 10% of methanol for 0.25 min before decreasing to 55% of 0.5 mM ammonium fluoride in water at 1 min, then decreasing to 40% of 0.5 mM ammonium fluoride in water at 6 min until 8.5 min, then decreasing to 0% of 0.5 mM ammonium fluoride in water at 10 min until 12.5 min, to a final of 90% of 0.5 mM ammonium fluoride in water and was kept at this for 1.99 min.

A seven-point calibration curve was used and calibration standards were analyzed alongside experimental samples. This involved an intermediate solution of 5 μg/mL of EE2 being prepared in methanol (LC-MS/MS grade, ≥99.9%). A water spike was then prepared in methanol at 0.01 µg/mL. Calibration standards were treated identically to experimental samples. No EE2 contaminations were detected in any control tanks throughout the exposure period. With this method, for EE2, a limit of quantification (LOQ) of 2 ng/L was obtained.

**Table S1.** Description of courtship behaviors in male halfbeaks (Greven 2010; Ogden et al. 2020).

| **Courtship behavior** | **Description** |
| --- | --- |
| Swimming under | Male aligns his head underneath the female’s genital pore (by less than one body height) so that he has visual access to the gravid spot. |
| Circling | Male approaches and swims around the female’s head in a semicircular direction. |
| Mating attempt | Male bends his body into a sigmoid (S-shaped) shape to facilitate contact between his andropodium (modified anal fin) and the female's genital pore. |

**S2. Sperm count**

Following completion of all sperm assays, samples were fixed in formalin by adding an equal volume of formalin to the sample with the largest sperm volume from each batch. Sperm concentration was then quantified (by SO) by loading 10 µL of the formalin-fixed sperm mixture into a Neubauer haemocytometer. Sperm cells were counted in five squares, and the mean count was used to calculate sperm concentration (cells per mL).

This measure was used as a proxy for sperm density and concentration and was included as a covariate in analyses of other sperm traits. Because sperm was not completely stripped from males, count was not treated as a primary response variable. In total, 73 males were included in the sperm counting.

**S3. Male length**

After completion of the behavioral trials, males were photographed (Canon EOS 800D; by RR and AR) in a photography tank (27 cm $\times$ 17.5 cm $\times$ 15.5 cm) filled with conditioned water. The fish were positioned sideways against the front of the tank using a small transparent photography chamber to ensure accurate lateral length measurements. The males were then humanely euthanized in a benzocaine solution (150 µL benzocaine per 1 mL ethanol). Using the photographs, ImageJ was used (by SO and AR) to measure body length as the distance from the anterior point of the eye to the caudal peduncle. Morphology was not a primary outcome of this experiment and was included only as a covariate in the analyses to account for potential size-related variation.

**S4. Statistical analysis**

Fixed effects included treatment (control, low EE2, high EE2) and trait-specific covariates. For sperm morphology, viability, and performance, we included male length (see S3 for male length methods), total count of mating behaviors, and mean sperm number per CASA field (local density, see S2 for sperm count methods) where relevant; for courtship counts, we included male length, mean female length, and mean gravid spot area, with female presentation order (large or small gravid spot first) added in the sequential assay, and the zero-inflation component of the courtship models included the same variables as in the main model formula. Preference models included gravid spot difference (difference in gravid spot area between large and small spot females), female body size difference, male length, and presentation order (in the sequential assay). Time to interact models were fitted including the same covariates as described for the preference models; however, for the sequential assay, the interaction between female size and presentation order was included instead of the ‘difference’ variables used in the preference models, and female length was included in the main model; the hurdle sub-model included treatment and male length (simultaneous assay), and treatment, relative spot size, and male length (sequential assay). In behavioral models, male length, female length, and gravid spot area were centered and scaled (mean = 0, SD = 1) to assist model fitting and interpretability of the results.

Random effects varied by model: tank identity was included for all single-response Gaussian models; and tank and male identity for the multivariate courtship model. For sperm morphology and sperm performance, we fitted joint multivariate models and estimated residual correlations among responses to assess trait covariation.

**Supporting results**

**S5. Analytical verification of EE2 concentrations**

Pairwise contrasts among model-estimated means confirmed that EE2 concentrations differed clearly between low and high treatments and between the sequential and simultaneous assays at the high EE2 level (Table S2). In contrast, measured EE2 concentrations did not differ meaningfully between the low treatments in the two experiments.

**Table S2.** Model-estimated differences in measured EE2 concentrations between treatments and experiments. Values are posterior median differences (ng/L) with 95% highest posterior density (HPD) intervals, derived from a Bayesian model fitted to tank-level EE2 concentrations.

| Contrast | Estimate | Lower 95% HPD | Upper 95% HPD |
| --- | --- | --- | --- |
| Simultaneous Low - Sequential Low | -78.86 | -173.99 | 20.15 |
| Sequential High - Sequential Low | 647.09 | 543.87 | 741.07 |
| Sequential High - Simultaneous Low | 726.12 | 625.34 | 822.61 |
| Simultaneous High - Sequential Low | 337.10 | 240.78 | 439.51 |
| Simultaneous High - Simultaneous Low | 416.40 | 318.86 | 515.36 |
| Simultaneous High - Sequential High | -309.87 | -407.31 | -211.98 |

**S6. Latency to interact with females**

In the sequential assay, males in both the low and high EE2 treatments initiated interactions later than control males, while low and high treatments were broadly comparable (Tables S3). The probability that a male did not initiate an interaction was low across treatments (Table S4). Covariates including male length and female length, showed no detectable influence on time to first interact (Table S5). Additional contrasts examining female presentation order and size were explored but showed no consistent or interpretable patterns across treatments and are therefore not reported.

In the simultaneous assay, males in the high EE2 treatment took longer to initiate interactions than males in the control and low EE2 treatments, which were similar to one another (Table S3). The probability that males did not interact with either female during the trial was uncertain across treatments (Table S4). No clear covariate effects were detected for male length, difference in female body size, or difference in female gravid spot size (Table S5).

**Table S3.** Observed and model-estimated mean latency to interact (sec) across treatments in the sequential and simultaneous assays. Observed means are calculated from males that interacted (>0 sec only), while model-estimated means represent posterior predictions conditional on an interaction occurring. Values are shown with 95% credible intervals (CrI).

| Experiment | Treatment | Observed mean (sec; >0 only) | Model-estimated mean (sec; conditional on interaction) | Lower 95% CrI | Upper 95% CrI |
| --- | --- | --- | --- | --- | --- |
| Sequential | Control | 118.71 | 107.56 | 68.63 | 151.62 |
| Sequential | Low | 217.50 | 211.50 | 143.53 | 288.38 |
| Sequential | High | 205.25 | 206.63 | 140.40 | 284.34 |
| Simultaneous | Control | 175.76 | 170.58 | 87.47 | 301.56 |
| Simultaneous | Low | 197.10 | 199.52 | 105.23 | 334.78 |
| Simultaneous | High | 483.34 | 471.43 | 239.08 | 802.77 |

**Table S4.** Posterior estimates of the probability that males did not initiate an interaction with either female during the trial. Values are model-estimated probabilities from the hurdle (zero) component of the model, with associated 95% credible intervals (CrI).

| Experiment | Treatment | Pr(no interaction) | Lower 95% CrI | Upper 95% CrI |
| --- | --- | --- | --- | --- |
| Sequential | Control | 0.00 | 0.00 | 0.02 |
| Sequential | Low | 0.04 | 0.00 | 0.11 |
| Sequential | High | 0.07 | 0.01 | 0.15 |
| Simultaneous | Control | 0.24 | 0.04 | 0.47 |
| Simultaneous | Low | 0.06 | 0.00 | 0.19 |
| Simultaneous | High | 0.09 | 0.00 | 0.23 |

**Table S5.** Posterior estimates of covariate effects on male latency to interact. Values are slopes on the response scale from the conditional mean (mu) component of the hurdle-gamma model, with associated 95% credible intervals (CrI). Negative slopes indicate shorter latencies to first interact, whereas positive slopes indicate longer latencies.

| Experiment | Covariate | Slope (response scale) | Lower 95% CrI | Upper 95% CrI |
| --- | --- | --- | --- | --- |
| Sequential | Male length | 0.13 | -0.09 | 0.35 |
| Sequential | Female length | -0.12 | -0.30 | 0.06 |
| Simultaneous | Male length | -0.01 | -0.27 | 0.25 |
| Simultaneous | Female body size difference | 0.04 | -0.50 | 0.56 |
| Simultaneous | Gravid spot difference | 0.11 | -0.73 | 1.01 |

**S7. Total occurrence of courtship behaviors**

In the sequential experiment, model-estimated mean total counts differed markedly among courtship behaviors, with swimming under and circling occurring more frequently than mating attempts across EE2 treatments (Table S6 and S7).

Continuous covariates showed limited and inconsistent associations with total courtship occurrence. Effects of male length, mean female length, and mean gravid spot area on swimming under, mating, and circling behaviors were generally weak, with credible intervals overlapping zero across behaviors (Table S8).

At the individual level, posterior correlations among male random effects revealed positive associations between swimming under behavior and both mating and circling behaviors, whereas the association between mating and circling behaviors was weaker and more uncertain (Table S9). Tank-level correlations among behaviors were low, indicating minimal shared variation attributable to housing tanks (Table S10).

In the simultaneous assay, model-estimated mean total counts varied among behaviors, with swimming under occurring substantially more frequently than mating or circling across all treatments (Table S6 and S7).

Continuous covariates showed limited influence on total courtship occurrence. Male length was positively associated with swimming under behavior, while effects of male length, mean female length, and mean gravid spot area on mating and circling behaviors were weak and not credibly different from zero (Table S8).

At the individual level, posterior correlations among male random effects revealed positive associations between swimming under and circling, whereas the association between mating and circling, and mating and swimming under was weaker and more uncertain (Table S9). Tank-level correlations among behaviors were low, indicating minimal shared variation attributable to housing tanks (Table S10).

**Table S6.** Observed and model-estimated mean total occurrences of male courtship behaviors. Values are shown for swimming under, mating, and circling behaviors across EE2 treatments. Model-estimated means and 95% credible intervals (CrI) are derived from Bayesian multivariate negative binomial models and are reported on the response scale. Estimated means represent expected total counts per trial, conditional on the fitted model.

| Experiment | Trait | Treatment | Observed mean | Estimated marginal mean | Lower 95% CrI | Upper 95% CrI |
| --- | --- | --- | --- | --- | --- | --- |
| Sequential | Mating | Control | 2.32 | 2.01 | 0.87 | 3.42 |
| Sequential | Mating | Low | 0.59 | 0.41 | 0.13 | 0.79 |
| Sequential | Mating | High | 0.41 | 0.20 | 0.02 | 0.56 |
| Sequential | Circling | Control | 14.23 | 11.31 | 6.70 | 17.54 |
| Sequential | Circling | Low | 12.85 | 9.09 | 5.57 | 13.66 |
| Sequential | Circling | High | 4.07 | 2.87 | 1.46 | 4.62 |
| Sequential | Swimming under | Control | 16.50 | 14.73 | 9.96 | 20.08 |
| Sequential | Swimming under | Low | 11.85 | 10.21 | 7.11 | 13.77 |
| Sequential | Swimming under | High | 9.15 | 6.46 | 4.31 | 9.07 |
| Simultaneous | Mating | Control | 0.38 | 0.17 | 0.02 | 0.42 |
| Simultaneous | Mating | Low | 0.42 | 0.23 | 0.04 | 0.56 |
| Simultaneous | Mating | High | 0.56 | 0.32 | 0.03 | 0.85 |
| Simultaneous | Circling | Control | 1.00 | 0.23 | 0.01 | 0.84 |
| Simultaneous | Circling | Low | 1.58 | 0.40 | 0.02 | 1.18 |
| Simultaneous | Circling | High | 0.48 | 0.22 | 0.01 | 0.82 |
| Simultaneous | Swimming under | Control | 15.75 | 9.38 | 4.03 | 16.83 |
| Simultaneous | Swimming under | Low | 15.29 | 9.45 | 4.71 | 15.76 |
| Simultaneous | Swimming under | High | 4.56 | 3.23 | 1.52 | 5.37 |

**Table S7.** Pairwise contrasts in total occurrences of male courtship behaviors. Estimates represent differences in expected total counts between EE2 treatments, calculated on the response scale from Bayesian multivariate negative binomial models. Credible intervals (95% CrI) that exclude zero indicate detectable treatment differences.

| Experiment | Behavior | Contrast | Estimate | Lower 95% CrI | Upper  95% CrI |
| --- | --- | --- | --- | --- | --- |
| Sequential | Swimming under | Control - Low | 4.44 | -1.44 | 10.55 |
| Sequential | Swimming under | Control - High | 8.20 | 2.86 | 14.09 |
| Sequential | Swimming under | Low - High | 3.73 | -0.27 | 8.01 |
| Sequential | Mating | Control - Low | 1.57 | 0.40 | 3.00 |
| Sequential | Mating | Control - High | 1.77 | 0.65 | 3.20 |
| Sequential | Mating | Low - High | 0.20 | -0.22 | 0.68 |
| Sequential | Circling | Control - Low | 2.19 | -4.19 | 9.43 |
| Sequential | Circling | Control - High | 8.34 | 3.52 | 14.58 |
| Sequential | Circling | Low - High | 6.15 | 2.34 | 11.10 |
| Simultaneous | Swimming under | Control - Low | -0.05 | -8.17 | 8.02 |
| Simultaneous | Swimming under | Control - High | 6.10 | 0.51 | 13.52 |
| Simultaneous | Swimming under | Low - High | 6.17 | 1.19 | 12.53 |
| Simultaneous | Mating | Control - Low | -0.06 | -0.41 | 0.28 |
| Simultaneous | Mating | Control - High | -0.14 | -0.73 | 0.23 |
| Simultaneous | Mating | Low - High | -0.08 | -0.67 | 0.35 |
| Simultaneous | Circling | Control - Low | -0.14 | -1.09 | 0.57 |
| Simultaneous | Circling | Control - High | 0.00 | -0.68 | 0.85 |
| Simultaneous | Circling | Low - High | 0.15 | -0.65 | 1.08 |

**Table S8.** Effects of continuous covariates on total courtship occurrences. Slopes are reported on the response scale from Bayesian multivariate negative binomial models and represent the expected change in total behavioral counts per unit increase in each covariate. Credible intervals (95% CrI) overlapping zero indicate no detectable covariate effect.

| Experiment | Behavior | Covariate | Slope (response scale) | Lower 95% CrI | Upper 95% CrI |
| --- | --- | --- | --- | --- | --- |
| Sequential | Swimming under | Male length | -2.08 | -4.72 | 0.51 |
| Sequential | Swimming under | Mean female length | 2.12 | -0.28 | 4.51 |
| Sequential | Swimming under | Mean gravid spot area | -1.54 | -5.15 | 2.23 |
| Sequential | Mating | Male length | -0.07 | -0.53 | 0.36 |
| Sequential | Mating | Mean female length | 0.11 | -0.32 | 0.57 |
| Sequential | Mating | Mean gravid spot area | 0.11 | -0.67 | 0.84 |
| Sequential | Circling | Male length | 0.60 | -2.10 | 3.24 |
| Sequential | Circling | Mean female length | 2.25 | -0.24 | 5.11 |
| Sequential | Circling | Mean gravid spot area | 0.93 | -3.24 | 4.84 |
| Simultaneous | Swimming under | Male length | 3.54 | 1.13 | 6.48 |
| Simultaneous | Swimming under | Mean female length | 1.12 | -2.24 | 4.68 |
| Simultaneous | Swimming under | Mean gravid spot area | -0.03 | -6.60 | 6.65 |
| Simultaneous | Mating | Male length | 0.08 | -0.07 | 0.27 |
| Simultaneous | Mating | Mean female length | -0.08 | -0.38 | 0.17 |
| Simultaneous | Mating | Mean gravid spot area | -0.18 | -0.83 | 0.34 |
| Simultaneous | Circling | Male length | 0.08 | -0.19 | 0.40 |
| Simultaneous | Circling | Mean female length | -0.22 | -0.83 | 0.13 |
| Simultaneous | Circling | Mean gravid spot area | 0.46 | -0.20 | 1.49 |

**Table S9.** Posterior mean correlations (r) and 95% credible intervals (CrI) among male-level random effects for total courtship behaviors.

| Experiment | Behavior pair | r | Lower 95% CrI | Upper 95% CrI |
| --- | --- | --- | --- | --- |
| Sequential | Swimming under – Circling | 0.75 | 0.47 | 0.96 |
| Sequential | Swimming under – Mating | 0.59 | -0.20 | 0.96 |
| Sequential | Mating – Circling | 0.40 | -0.35 | 0.89 |
| Simultaneous | Swimming under – Circling | 0.70 | 0.12 | 0.97 |
| Simultaneous | Swimming under – Mating | 0.66 | -0.19 | 0.98 |
| Simultaneous | Mating – Circling | 0.59 | -0.23 | 0.96 |

**Table S10.** Posterior mean correlations (r) and 95% credible intervals (CrI) among tank-level random effects for total courtship behaviors.

| Experiment | Behavior pair | r | Lower 95% CrI | Upper 95% CrI |
| --- | --- | --- | --- | --- |
| Sequential | Swimming under – Circling | 0.10 | -0.85 | 0.91 |
| Sequential | Mating – Circling | 0.00 | -0.88 | 0.88 |
| Sequential | Swimming under – Mating | -0.02 | -0.88 | 0.86 |
| Simultaneous | Swimming under – Circling | 0.16 | -0.79 | 0.91 |
| Simultaneous | Mating – Circling | 0.05 | -0.86 | 0.89 |
| Simultaneous | Swimming under – Mating | 0.03 | -0.87 | 0.89 |

**S8. Preference**

In both the sequential and simultaneous assays, there was no evidence that EE2 exposure affected male preference for female gravid spot size. In the sequential assay, model-estimated mean preference values were similar across treatments, with credible intervals overlapping broadly among control, low EE2, and high EE2 males. Pairwise treatment contrasts expressed as odds ratios showed substantial uncertainty, with all credible intervals overlapping one (Table S11). In the simultaneous assay, preference estimates likewise overlapped across EE2 treatments, and no pairwise contrasts indicated detectable treatment differences (Table S11).

Continuous covariates showed no consistent associations with preference in either assay. Effects of male length, gravid spot size difference, and female body size difference were weak, with slope credible intervals overlapping zero (Table S12). Exploratory analyses of female presentation order in the sequential assay showed no clear influence on preference and are not reported.

**Table S11.** Pairwise treatment contrasts in preference, quantified as the proportion of swimming under behavior directed toward the female with the larger gravid spot. Contrasts are reported as odds ratios (OR) from Bayesian models on the response scale. OR values greater than 1 indicate higher odds of directing swimming under behavior toward the large gravid-spot female in the first treatment level of the contrast. Credible intervals (95% CrI) overlapping 1 indicate no detectable treatment difference.

| Experiment | Contrast | Odds ratio | Lower 95% CrI | Upper 95% CrI |
| --- | --- | --- | --- | --- |
| Sequential | Low / Control | 1.21 | 0.26 | 3.01 |
| Sequential | High / Control | 1.13 | 0.28 | 2.71 |
| Sequential | High / Low | 0.93 | 0.21 | 2.26 |
| Simultaneous | Low / Control | 1.82 | 0.73 | 3.37 |
| Simultaneous | High / Control | 1.55 | 0.54 | 3.08 |
| Simultaneous | High / Low | 0.85 | 0.30 | 1.68 |

**Table S12.** Effects of continuous covariates on preference. Slopes are reported on the response scale from Bayesian models and represent the expected change in preference for the large gravid-spot female per unit increase in each covariate. Positive slopes indicate increased preference for the large gravid-spot female. Credible intervals (95% CrI) overlapping zero indicate no detectable covariate effect.

| Experiment | Covariate | Slope (response scale) | Lower 95% CrI | Upper 95% CrI |
| --- | --- | --- | --- | --- |
| Sequential | Male length | -0.13 | -0.57 | 0.26 |
| Sequential | Gravid spot difference | -0.04 | -0.37 | 0.32 |
| Sequential | Female body size difference | 0.20 | -0.32 | 0.70 |
| Simultaneous | Male length | 0.12 | -0.17 | 0.40 |
| Simultaneous | Gravid spot difference | 0.15 | -0.67 | 1.00 |
| Simultaneous | Female body size difference | 0.12 | -0.36 | 0.55 |

**S9. Sperm morphology**

EE2 exposure did not affect sperm morphology (Figure S1). Model-estimated marginal means for sperm head length, midpiece length, and total sperm length broadly overlapped across treatments, with differences small relative to within-treatment variability.

Posterior mean correlations were small in magnitude, and credible intervals overlapped zero for all trait pairs, indicating no strong residual covariation among sperm morphology traits after accounting for fixed and random effects (Table S13).

**Figure S1.** Plot showing sperm head, midpiece and total length (µm), plotted by exposure treatment, with control fish represented by blue (*n* = 23), low exposure treatment in orange (*n* = 25) and high exposure treatment in red (*n* = 25). The colored area shows the probability density of the raw data at different values smoothed by a kernel density estimator. Points inside the colored area represent jittered raw data, and solid diamonds are model-estimated marginal means, with error bars showing 95% credible intervals.

**Table S13.** Posterior summaries of residual correlations (rescor) among sperm morphology traits from the multivariate Bayesian model. Residual correlations quantify trait–trait associations after accounting for fixed effects (including treatment) and random effects (including tank). Correlations are unitless and range from −1 to +1. Credible intervals (95% CrI) overlapping zero indicate no detectable residual association.

| Trait Pair | Estimate | Est.Error | Lower 95% CrI | Upper 95% CrI |
| --- | --- | --- | --- | --- |
| Midpiece – Sperm total length | 0.22 | 0.12 | -0.02 | 0.45 |
| Head – Sperm total length | -0.22 | 0.13 | -0.45 | 0.06 |
| Head – Midpiece | -0.12 | 0.15 | -0.39 | 0.18 |

**S10. Sperm viability**

EE2 exposure did not affect sperm viability (Figure S2). Model-estimated marginal means of the proportion of live sperm broadly overlapped across treatments, with differences small relative to within-treatment variability.

**Figure S2.** Plot showing sperm viability (proportion of live sperm), plotted by exposure treatment, with control fish represented by blue (*n* = 23), low exposure treatment in orange (*n* = 25) and high exposure treatment in red (*n* = 25). The colored area shows the probability density of the raw data at different values smoothed by a kernel density estimator. Points inside the colored area represent jittered raw data, and solid diamonds are model-estimated marginal means, with error bars showing 95% credible intervals.

**S11. Sperm performance**

There was no strong residual correlation between sperm curvilinear velocity (VCL) and linearity after accounting for fixed and random effects, with the posterior mean correlation small and its credible interval overlapping zero (Table S14). Consequently, sperm velocity and linearity were interpreted independently.

EE2 treatment had no detectable effect on sperm performance (Figure S3). Model-estimated marginal means for both VCL and linearity broadly overlapped across treatments, with differences small relative to within-treatment variability.

**Table S14.** Posterior summary of the residual correlation (rescor) between sperm curvilinear velocity (VCL) and sperm linearity (LIN) from the multivariate Bayesian model. The residual correlation quantifies the association between traits after accounting for fixed effects (including treatment and covariates) and random effects (including tank). Correlations are unitless and range from −1 to +1; credible intervals (95% CrI) overlapping zero indicate no detectable residual association.

| Trait Pair | Estimate | Est.Error | Lower 95% CrI | Upper 95% CrI |
| --- | --- | --- | --- | --- |
| VCL mean – Linearity mean | 0.03 | 0.13 | -0.21 | 0.28 |

**Figure S3.** Plot showing sperm performance (velocity and linearity), plotted by exposure treatment, with control fish represented by blue (*n* = 22), low exposure treatment in orange (*n* = 25) and high exposure treatment in red (*n* = 25). The colored area shows the probability density of the raw data at different values smoothed by a kernel density estimator. Points inside the colored area represent jittered raw data, and solid diamonds are model-estimated marginal means, with error bars showing 95% credible intervals.

**References**

Greven, H. What do we know about reproduction of internally fertilizing halfbeaks (Zenarchopteridae)? In Viviparous Fishes II; Uribe, M. C.; Grier, H. J., Eds.; New Life Publications: Homestead, FL, 2010; pp 121–141.

Ogden, H. J. P.; De Boer, R. A.; Devigili, A.; Reuland, C.; Kahrl, A. F.; Fitzpatrick, J. L. Male Mate Choice for Large Gravid Spots in a Livebearing Fish. *Behav. Ecol.* **2020**, *31* (1), 63–72; DOI 10.1093/BEHECO/ARZ156.
